# Epigenetic regulation of chemokine (CC‐motif) ligand 2 in inflammatory diseases

**DOI:** 10.1111/cpr.13428

**Published:** 2023-03-05

**Authors:** Yingyi Chen, Siyan Liu, Lili Wu, Yitong Liu, Juan Du, Zhenhua Luo, Junji Xu, Lijia Guo, Yi Liu

**Affiliations:** ^1^ Laboratory of Tissue Regeneration and Immunology and Department of Periodontics, Beijing Key Laboratory of Tooth Regeneration and Function Reconstruction, School of Stomatology Capital Medical University Beijing People's Republic of China; ^2^ Immunology Research Center for Oral and Systemic Health, Beijing Friendship Hospital Capital Medical University Beijing People's Republic of China; ^3^ Department of Orthodontics, School of Stomatology Capital Medical University Beijing People's Republic of China

## Abstract

Appropriate responses to inflammation are conducive to pathogen elimination and tissue repair, while uncontrolled inflammatory reactions are likely to result in the damage of tissues. Chemokine (CC‐motif) Ligand 2 (CCL2) is the main chemokine and activator of monocytes, macrophages, and neutrophils. CCL2 played a key role in amplifying and accelerating the inflammatory cascade and is closely related to chronic non‐controllable inflammation (cirrhosis, neuropathic pain, insulin resistance, atherosclerosis, deforming arthritis, ischemic injury, cancer, etc.). The crucial regulatory roles of CCL2 may provide potential targets for the treatment of inflammatory diseases. Therefore, we presented a review of the regulatory mechanisms of CCL2. Gene expression is largely affected by the state of chromatin. Different epigenetic modifications, including DNA methylation, post‐translational modification of histones, histone variants, ATP‐dependent chromatin remodelling, and non‐coding RNA, could affect the ‘open’ or ‘closed’ state of DNA, and then significantly affect the expression of target genes. Since most epigenetic modifications are proven to be reversible, targeting the epigenetic mechanisms of CCL2 is expected to be a promising therapeutic strategy for inflammatory diseases. This review focuses on the epigenetic regulation of CCL2 in inflammatory diseases.

## Graphical abstract

This review described the epigenetic regulation of CCL2 and described the crosstalk between metabolic reprogramming and CCL2 epigenetic regulation in the inflammatory environment. In chronic inflammation, CCL2 plays an indispensable role in the pathogenesis of inflammatory progression and excessive fibrosis. Epigenetic regulation is essential to accurately adjust the transcription of target genes. Various epigenetic modifications are capable to change the ‘open’ or ‘closed’ state of chromatin and thus alter the transcriptional activity of target genes. In this review, we discussed the epigenetic regulation of CCL2 in inflammatory diseases aiming to provide promising strategies for the treatment of non‐controllable inflammation.

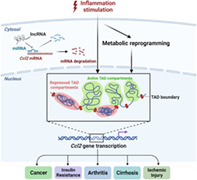

## 1 BACKGROUND

When tissues are suffered from infection or injury, chemokines play a crucial role in the progress of recruiting immune cells into the inflammation area to eliminate pathogens and repair tissues.^1^ Chemokines are signalling proteins whose molecular weights were small (8–14 kDa).^2^ Chemokines have been divided into the following four subfamilies according to the sequence characteristics of cysteine residues: the CC, CXC, CX3C and XC.^3^, ^4^, ^5^ In inflammatory conditions, the imbalance of chemokines was proven to be involved in the pathogenesis of a variety of inflammation diseases, such as cardiovascular diseases, arthritis, neuropathic pain, cancer, and so on.^2^, ^6^, ^7^ This disorder of chemokines could lead to abnormalities in the process of tissue repair, such as delayed wound healing or excessive fibrosis, which was related to various diseases, including diabetic foot, kidney fibrosis, cirrhosis, and so on.^8^, ^9^, ^10^ These uncontrolled inflammatory states caused by the disorder of chemokines are still a difficult problem in clinical treatment.

CC‐chemokine group is the largest subfamily of chemokines and is characterized by two adjacent cysteines at the amino terminus of the protein.^11^, ^12^ Among them, CC‐chemokine ligand 2 (CCL2, also known as monocyte chemokine protein‐1, MCP‐1) is the first chemokine to be purified and extensively studied. It is the main chemokine and activator of monocytes, macrophages, and neutrophils.^13^ Under normal circumstances, appropriate responses of inflammation are conducive to pathogen elimination and tissue repair, while excessive inflammation responses are likely to result in the damage of tissues.^14^ After that, cells in the injury site would express CCL2 to recruit and activate immune cells in the inflammatory area, while the infiltrated inflammatory cells could further secrete CCL2 to amplify and accelerate the inflammatory cascade.^15^, ^16^ In recent years, studies have reported that CCL2 is closely related to chronic non‐controllable inflammation, such as cirrhosis, neuropathic pain, insulin resistance, atherosclerosis, deforming arthritis, ischemic injury, cancer, and so on.^17^, ^18^, ^19^, ^20^, ^21^, ^22^, ^23^, ^24^, ^25^ Given the important regulatory role of chemokines in inflammation, an in‐depth understanding of the regulatory mechanism of CCL2 is conducive to providing new therapeutic strategies for inflammatory diseases.

In recent years, many studies have explored the regulatory mechanism of CCL2 expression. Specifically, oxidative stress injury and virus infection could up‐regulate the expression of CCL2 via NF‐ĸB signalling pathways and lead to excessive fibrosis or inflammatory cascade.^26^, ^27^ In the tumour immune microenvironment, proteasome activation factor PA28γ could activate the NF‐ĸB signalling pathway of oral squamous carcinoma (OSCC) cells and enhance the expression of CCL2, which promote tumour angiogenesis and reduce the survival rate.^28^ In addition, HMGA2, a tumour derivative, could up‐regulate the gene transcription of CCL2 in tumour‐associated macrophages (TAMs) by regulating the STAT3 signalling pathway. In the state of CCL2 disorder, macrophage recruitment into the microenvironment of tumour and result in distant metastasis and reduced survival rate.^29^ Moreover, in desmoplasia‐associated cancers, fibroblast activation protein (FAP) could up‐regulate the expression of CCL2 through STAT3 signalling pathway, which enhances the recruitment of myeloid‐derived suppressor cells and promotes proliferation of tumour matrix and leads to poor prognosis.^30^ From these evidences, activation of multiple signalling pathways in inflammatory diseases, such as oxidative stress, infection, or tumour, ultimately leads to transcription factors transporting into the nucleus and enhancing the gene transcription of CCL2. However, chromatin is made up of DNA, RNA, and histones in a tightly coiled structure. This creates a physical barrier to gene transcription.^31^, ^32^ Therefore, it is indispensable to investigate the accessibility of chromatin while studying the regulatory mechanism of CCL2.

In a recent couple of years, the regulatory roles of epigenetics on a variety of inflammatory diseases such as pro‐neoplastic inflammation, neuropathic pain, pulpitis, and periodontitis have gradually attracted attention.^33^, ^34^, ^35^ Epigenetics is defined as the heritable changes in gene functions that ultimately lead to phenotypic changes without changes in the DNA sequence.^36^ When tissues are challenged by injury or inflammation, innate and adaptive immune cells need to quickly adjust the transcription of a large number of gene‐encoded cytokines in response to the specific immune environment. The precise regulation of this progress is based on epigenetic reprogramming by altering the transcriptional state of immune response‐related cytokines.^37^ Gene expression is largely affected by the state of chromatin. Different epigenetic modifications could affect the ‘open’ or ‘closed’ state of DNA. The ‘open’ state of DNA could allow transcription mechanisms or transcription factors to bind with it and promote effective and powerful activation of transcription.^37^ The loose/compressive state of chromatin is dynamic and is regulated by multiple mechanisms including DNA methylation, post‐translational modification (PTM) of histones (e.g., Lysine phosphorylation, acetylation, methylation, ubiquitination, lactation, etc.), histone variants, ATP‐dependent chromatin remodelling and non‐coding RNA (ncRNA).^38^, ^39^, ^40^ Among them, histone post‐transcriptional modification, DNA methylation and ncRNA have been reported to be involved in the epigenetic regulation of CCL2.^34^, ^41^, ^42^ Since most epigenetic modifications are proven to be reversible, epigenetic modification targeting CCL2 is expected to be a promising therapeutic strategy for inflammatory diseases. This review focuses on the epigenetic regulation of CCL2 in inflammatory diseases.

## 2 HISTONE MODIFICATIONS MEDIATED REGULATION OF CCL2 IN INFLAMMATORY DISEASES

**FIGURE 1:**
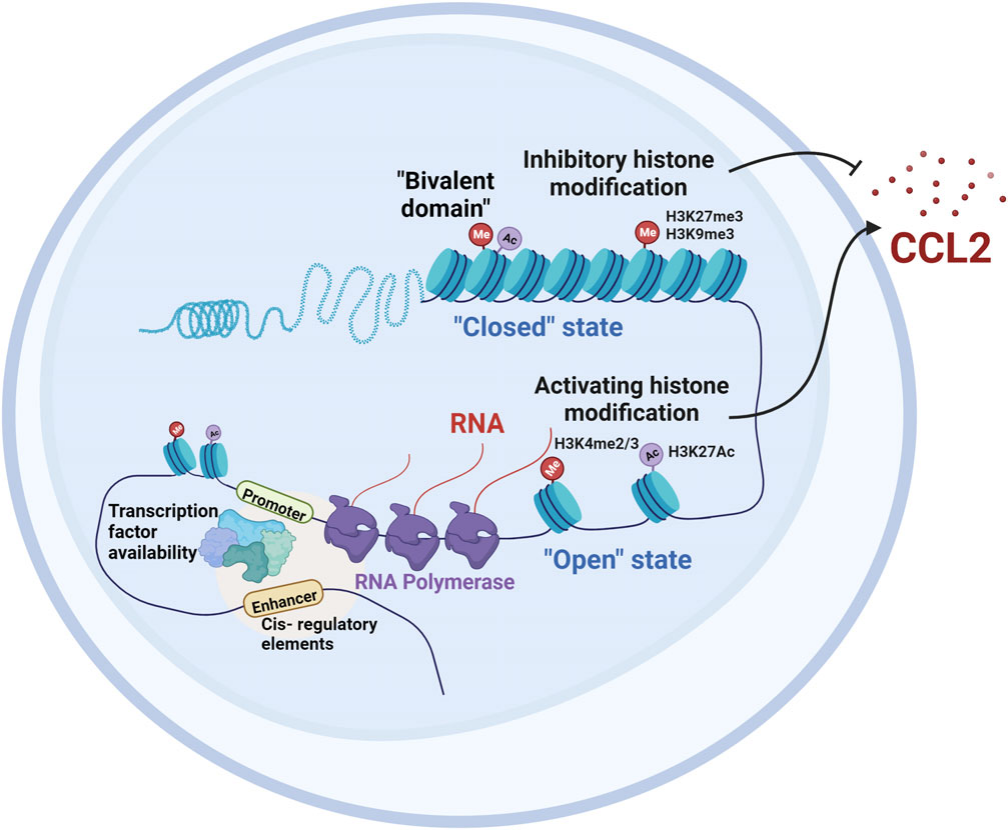
Histone modifications mediated regulation of CCL2 in inflammatory diseases. Created with BioRender.com.

PTM of histone modifications could influence the initiation and elongation of transcription by affecting the loose/compressive state of chromatin. PTMs of histones include histone phosphorylation, acetylation, methylation and ubiquitination.^43^, ^44^, ^45^, ^46^ Most of the PTMs of histones are reversible and involve a variety of enzymes, such as lysine acetyltransferases (KATs, also known as histone acetylated transferases [HATs]), and lysine deacetylases (KDAC, also known as histone deacetylase [HDACs]), histone lysine methyltransferase (KMT) and histone lysine demethylases (KDM), ubiquitination enzyme (E1, E2, and E3 enzymes) and deubiquitination enzyme (DUB), and mitogen‐ and stress‐activated protein kinases 1/2 (MSK1/2).^43^, ^46^, ^47^, ^48^, ^49^, ^50^, ^51^ These enzymes are typically present in multisubunit complexes and then modify specific residues in the n‐terminal tail region or the globular domain of the core histones (H2A, H2B, H3, and H4). For example, EZH2, the catalytic subunit of the polycomb repressor complex 2 (PRC2), tri‐methylates lysine residue 27 of histone H3 (H3K27me3).^38^ In inflammatory diseases, the activating histone modifications of CCL2 include H3K27Ac, H3K4me2/3, H3Ser10 Phosphorylation; inhibitory histone modifications include H3K27me3, H3K9me3, and so on (Figure 1).^41^, ^52^, ^53^, ^54^

Methyltransferase METTL3 in non‐alcoholic steatohepatitis (NASH) could induce the deacetylation of H3K27 and H3K9 by recruiting HDAC1/2 on the CCL2 promoter, thus inhibiting the expression of CCL2 and inhibiting the progression of NASH.^55^ Consistently, in the case of obesity, CCL2 expression is up‐regulated when H3K27Ac enrichment is occupied on the CCL2 promoter of macrophages. In addition, Kiguchi et al.^56^ found that in the chronic inflammation caused by peripheral nerve injury, the level of H3K9Ac on the CCL2 promoter of the damaged sciatic nerve was increased, which increase the transcriptional activity of CCL2. Similarly, H3K4me3, an activating histone modification, was detected in Hashimoto's thyroiditis to enrich the promoter region of CCL2 and resulted in persistent up‐regulation of CCL2 expression of thyroid follicular cells. Han et al.^57^ confirmed that puerarin (a kind of isoflavone glycoside in Pueraria lobata) was capable to restrain the high‐glucose‐induced CCL2 expression by inhibiting histone 3 lysine 4‐methylation (H3K4me2/3) on the promoter of CCL2 in endothelial cells. Thus, puerarin has therapeutic potential in diabetes‐induced microcirculatory dysfunction.

In contrast to the activation effect, EZH2 (H3K27 methyltransferase) could mediate the inhibitory epigenetic modification of H3K27 tri‐methylation of CCL2 and could repress CCL2 expression of macrophages. In highly invasive and fatal small cell lung cancer, this effect could result in reduced infiltration of innate immune cells in the tumour and promote the progression of cancer.^58^ Interestingly, EZH2 could directly occupy the promoter of CCL2 and up‐regulate the expression of pro‐inflammatory cytokines and chemokines in mouse lupus‐like disease and human pulp cells.^59^, ^60^ These results were different from previously reported inhibitory effects of H3K27me3. The reason may be related to the interaction of multiple epigenetic modifications on the promoter, but further experiments are needed to investigate. In addition, the regulatory roles of CCL2 expression by other types of histone modifications have also been reported, including histone phosphorylation, ubiquitination, crotonylation, and so on.^45^, ^61^ Among them, Ruiz‐Andres et al. reported for the first time in 2016 that histone crotonylation could reduce the expression of CCL2 in acute kidney injury and has therapeutic potential for acute kidney injury.^62^

Interestingly, in recent years, researchers have found that histone modifications regulate inflammatory diseases with both inhibitory and activating characteristics. Bernstein et al. first identified this particular structure in embryonic stem cells in 2006 and named these seemingly opposite histone markers the ‘bivalent domain’. The existence of ‘bivalent domain’ enables gene expression to be rapidly activated under numerous stimulations in the environment.^63^ At first, the discussion of this special structure was mainly about its role in cell differentiation and maintenance of stem cell pluripotency.^13^, ^64^, ^65^, ^66^, ^67^, ^68^, ^69^ In 2016, Qiao et al. demonstrated the effectiveness of the ‘bivalent domain’ characterized by H3K27Ac/H3K27me3 labelling (H3K27Ac is associated with gene activation and H3K27me3 is associated with gene silencing) could regulate the function of human macrophages in inflammatory diseases.^53^, ^70^ Later, Yong et al. also revealed the existence of a ‘bivalent domain’ characterized by H3K4me3/H3K27me3 on Traf2 and Traf5 promoters. The decrease of EZH2 could mediate the loss of inhibitory markers and the disruption of the equilibrium state of the ‘bivalent domain’, so that TRAF2/5 enhanced NF‐κB signalling, and thus significantly increased the expression levels of TNF‐α, IL‐6, CCL2, and other inflammatory factors.^53^ In conclusion, a variety of histone modifications alters the transcription activity of CCL2 and may provide potential targets for the treatment of inflammatory diseases.

## 3 DNA METHYLATION‐MEDIATED REGULATION OF CCL2 IN INFLAMMATORY DISEASES

**FIGURE 2:**
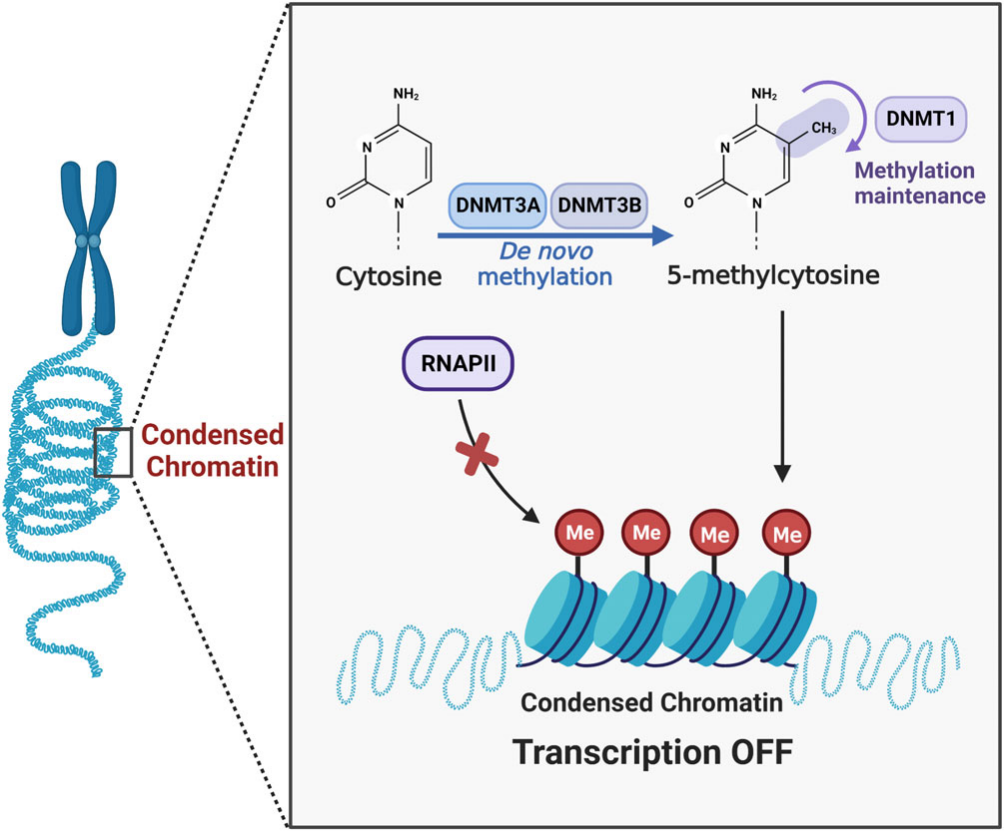
DNA methylation‐mediated regulation of CCL2 in inflammatory diseases. Created with BioRender.com.

DNA methylation occurs only on the cytosine (CpG dinucleotide) before guanine in the sequence of DNA. In this reaction, S‐adenosyl‐methionine (SAM) is used as a methyl donor, and one methyl group would be added to the cytosine ring under the catalysis of DNA methyltransferases (DNMT) to form methylcytosine. Four DNMTs have been found to share a conserved DNMT domain. DNMT1 can maintain DNA methylation during replication, DNMT3a and DNMT3 are responsible for catalysing new methylation, while DNMT2 only has a weak activity.^71^ The DNA methylation on the promoter is often associated with gene silencing. Two models of DNA methylation inhibiting gene expression have been proposed: firstly, cytosine methylation could directly prevent the binding of some transcription factors; Secondly, DNA methylation indirectly affects chromatin status (MBP) through the recruitment of methyl CpG binding proteins^72^, ^73^, ^74^ (Figure 2).

DNA methylation is thought to be the regulatory mechanism behind some inflammatory processes. The physiological effects of methyl donors (e.g., folate, choline, etc.) on inflammatory diseases are related to the synthesis of SAM, which is the universal methyl donor. For example, folic acid could alleviate the LPS‐induced inflammatory response of macrophages and could be helpful to alleviate various inflammation‐related diseases.^75^ Cui et al.^76^ also confirmed that folic acid could modify DNA methylation through methionine cycling, which improved the activity of DNMTs in the CCL2 promoter region and inhibited the expression of CCL2, thus effectively preventing atherosclerosis. In addition, DNA methylation also participates the regulatory mechanism of promoting tumour inflammation in which macrophages are important cellular components. However, CCL2 is responsible for inducing macrophages to play a pro‐tumour role. In several kinds of human tumours, PTX3 expression is inhibited by methylation of promoter and enhancer regions of human n‐pentaginin‐3, an important component of humoral immunity in innate immunity. PTX3 deficiency further leads to the up‐regulation of CCL2 expression and promotes tumour recruitment of macrophages. Therefore, PTX3, under the regulation of DNA methylation, could affect the expression of CCL2 and further regulate pro‐tumour inflammation.^77^

Importantly, Aoi et al.^78^ found that under hypoxia conditions, the levels of DNA methylation and histone H3K9 dimethylation in CCL2 enhancer and promoter region were significantly increased and jointly inhibited the expression of IL‐1β‐induced MCP‐1 under hypoxia conditions. This discovery revealed the epigenetic mechanism of the inhibition regulation of CCL2 under hypoxia, as well as the functional correlation between DNA methylation and histone methylation in gene silencing. The close relationship between them has also been confirmed by many studies.^79^, ^80^ Some researchers believed that there might be a mutually reinforcing and self‐circulating epigenetic network between DNA methylation, deacetylation and methylation of H3K9, ultimately leading to long‐term inhibition of gene expression.^81^ In addition, it is important to note that although DNA methylation is usually associated with gene suppression, it may also play an activation role of gene transcription. Petrus et al.^61^ found that reduced expression of SLC19A1 (a gene encoding membrane folate carrier) in human adipose cells induced hypermethylation on the CCL2 promoter, leading to increased expression of CCL2. This dysfunction of adipocytes leads to inflammation and insulin resistance in white adipose tissue through an epigenetic pathway.

## 4 NCRNA‐MEDIATED REGULATION OF CCL2 IN INFLAMMATORY DISEASES

### 4.1 MicroRNA regulation of CCL2 in inflammatory diseases

**FIGURE 3:**
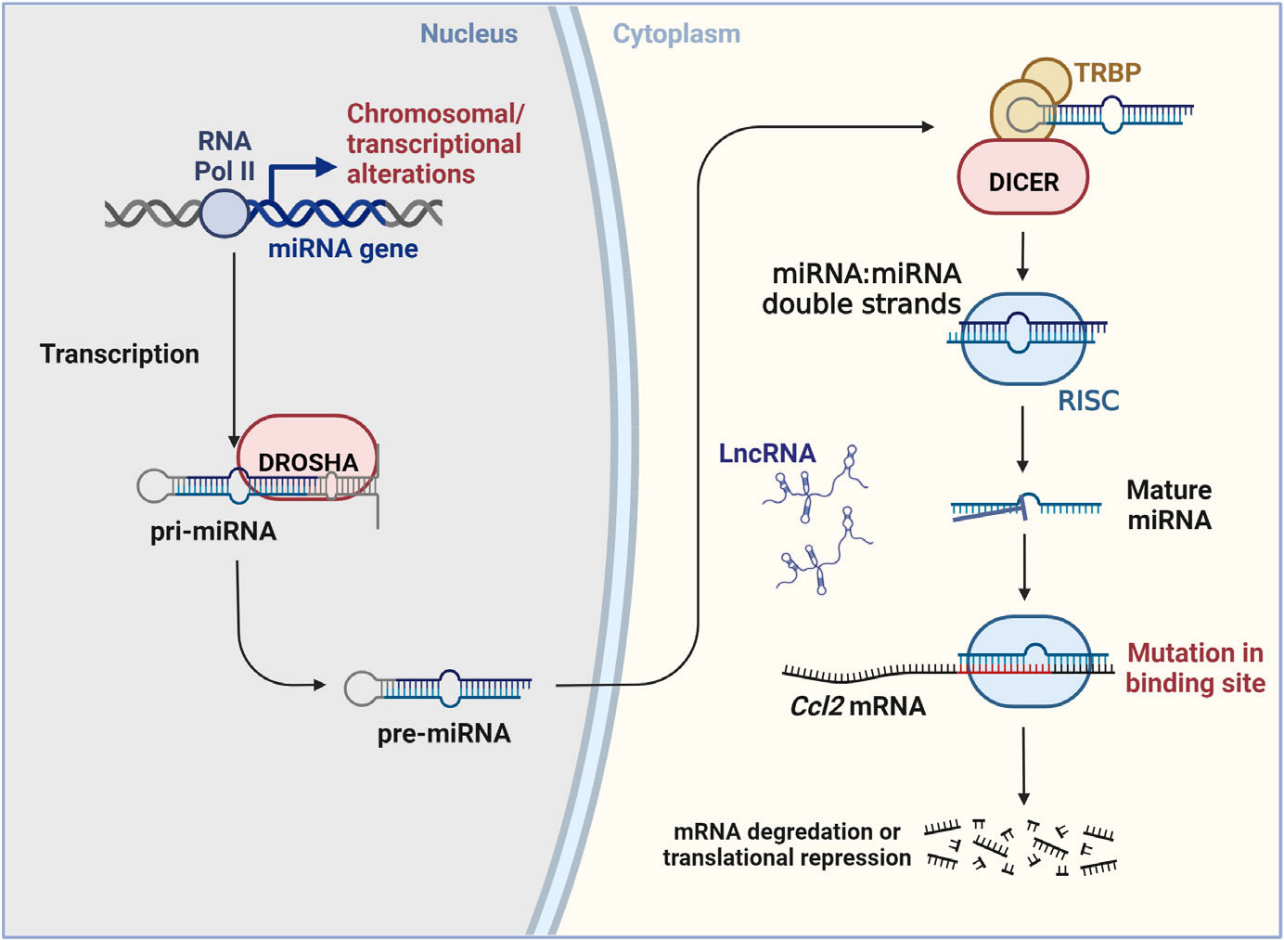
Non‐coding RNA (miRNA and lncRNA)‐mediated regulation of CCL2 in inflammatory diseases. Created with BioRender.com.

The microRNA (miRNA), a kind of ncRNA, could negatively regulate the expression of target genes at the post‐transcriptional level. The primary transcript of miRNA is very long, which is processed into miRNA precursors with hairpin structures in the nucleus and then transported to the cytoplasm. Subsequently, miRNA precursors are cut by Dicer into miRNA: miRNA double‐stranded with 20–25 nucleic acid lengths, and combined with Argonaute family proteins to form an RNA‐induced silencing complex (RISC). One of the miRNA chains is eventually degraded, and only one single miRNA chain is retained in RISC to fully or incompletely bind to the target mRNA to achieve degradation or translation inhibition of the target mRNA.^82^ miRNA not only plays a key role in physiological processes such as cell development, proliferation, and apoptosis, but also participates in the regulation of many pathological processes (Figure 3).

In inflammatory diseases, a variety of miRNAs are related to the regulation of CCL2 expressions, such as miR‐146b, miR‐125a‐5p, miRNA‐325‐5p, miR‐374, and so on. Hypoxia could induce the expression of tumour necrosis factor receptor‐associated factor 6 (TRAF6) and its downstream pro‐inflammatory factors IL‐6 and CCL2 by inhibiting miR‐146b. This hypoxia‐induced miR‐146b‐TRAF6‐IL‐6/CCL2 axis may induce cardiac fibrosis, dysfunction and even heart failure through the epigenetic pathway.^83^ However, in type 1 diabetes (T1D), the deficiency of regulatory T cells (Treg) plays an important role. Sebastiani et al. found that in the pancreatic draining lymph nodes of T1D patients, the specific high expression of miR‐125a‐5p in Treg cells led to the reduced expression of the target gene CCL2, thus limiting the migration and function of Treg cells in the pancreas.^84^

Pain is one of the signs of inflammation and may play the role of protection, in the acute or chronic phase of inflammation. However, chronic pain brings great agony to patients. But current treatments for painful conditions have limited efficacy.^85^ For pain‐related diseases, CCL2 plays a key role in the peripheral mechanism of neuropathic pain. Studies have shown that the expression of CCL2 in damaged DRG is up‐regulated at the protein and gene levels,^86^, ^87^ and is released from DRG neurons in a calcium‐dependent manner, which directly triggers an action potential in DRG neurons after selective binding with its receptor CCR2.^88^ At present, studies have gradually paid attention to the important role of miRNA in pain‐related diseases. In investigating the mechanism of miRNA‐325‐5p in chronic visceral pain, Wui et al.^89^ found that the expression of miRNA‐325‐5p was suppressed in the rat model of neonatal colonic inflammation (NCI), which was a neonatal model of chronic visceral pain. This suppression of miRNA‐325‐5p led to increased expression of CCL2 in the colon‐associated dorsal root ganglion. Intrathecal injection of miRNA‐325‐5p agomir (a chemically modified miRNA agonist) could significantly inhibit the up‐regulation of CCL2 expression in NCI rats. However, the application of Bindarit (an inhibitor of CCL2), significantly increased the threshold of colorectal dilation pain in NCI rats in a dose and time‐dependent manner. This suggests that NCI up‐regulates the expression of CCL2 by inhibiting the expression of miRNA‐325‐5p, thus leading to the high sensitivity of rat viscera. Similarly, some researchers found that miR‐374 was down‐regulated in painful endometriosis tissues, while the expression level of CCL2 targeted by Mir‐374 was significantly increased.^17^ Based on the inhibitory effect of multiple miRNAs on CCL2, targeted regulation of pain‐related miRNA expression is expected to be a promising treatment strategy for pain diseases.

### 4.2 Regulation of long ncrna of CCL2 in inflammatory diseases

Long non‐coding RNAs (lncRNAs), longer than 200 nucleotides, have no protein‐coding ability and exist both in the nucleus and cytoplasm of cells. LncRNAs could regulate mRNA transcription by cis or transaction. LncRNAs exhibit their function with multiple regulatory mechanisms, such as interacting with RNA, DNA and specific proteins (such as chromatin remodelling complex, transcription factors, RNA binding protein, etc.).^90^ Epidemiological and laboratory evidence suggested the correlation and therapeutic potential of CCL2/CCR2 with the pathogenesis of atherosclerosis, and there is increasing evidence that lncRNAs are misregulated in atherosclerosis.^91^ Based on this, a study of Nadiya et al.^91^ found that *lncRNA‐CCL2*, as a kind of lncRNA, was able to promote the expression of CCL2 in vascular endothelial cells, and the expression of *lncRNA‐CCL2* is associated with atherosclerotic plaques with unstable symptoms. The Pull‐down experiment further verified that the mechanism of *lncRNA‐CCL2* was to regulate the expression of CCL2 and promote atherosclerosis through the interaction with RNA‐binding proteins.

Studies have confirmed that LncRNA disorders also exist in painful diseases.^92^, ^93^ Nerve injury‐specific lncRNAs (*NIS‐lncRNAs*) are expressed in injured DRGs. *NIS‐lncRNAs* promote CCL2 transcription and trigger neuropathic pain by interacting with DNA/RNA‐binding proteins in DRG neurons.^94^ Interestingly, lncRNAs could regulate the expression of CCL2 by interacting with other epigenetic mechanisms. Studies have shown that the expression of lncRNA in Schwann cells and their exosomes, called regenerative associated transcription factor (*lncARAT*), was up‐regulated after sciatic nerve injury. *lncARAT* was shown that contributed to axon regeneration and functional recovery. Importantly, on the one hand, *lncARAT* was able to trimethylate H3K4 at the promoter of CCL2 by raising KMT2A, promoting the CCL2 expression of Schwann cells and recruiting macrophages to the injured site. Furthermore, infiltrating macrophages could phagocytose the exosomes of Schwann cells which contained a high expression of lncARAT. The lncARAT could combine with the miRNA‐329‐5p in macrophages, then the expression of suppressor of cytokine signalling (SOCS) 2 would be up‐regulated. The repair function of would be promoted through the STAT1/6‐dependent pathway.^95^ In conclusion, *lncRNAs*, as a class of heterogeneous molecules, have multiple regulatory effects on the expression of CCL2 in macrophages inflammatory diseases, and could also exert their regulatory effects by acting on other epigenetic mechanisms (histone modification or miRNA, etc.) (Figure 3).

## 5 METABOLIC‐EPIGENOMICS REGULATION OF CCL2

In the process of inflammation, immune cells would undergo metabolize reprogramming under the challenge of pathogenic microorganisms and inflammation.^96^ Cell metabolism has a significant impact on the functions of immune cells. For example, in the process of macrophage polarization or T cell activation, to deal with the biosynthesis needs of macromolecular, glucose metabolism would change from oxidative phosphonic acidification (efficient but relatively slow) to glycolysis (rapid energy supply).^25^, ^97^ In the process of metabolic reprogramming, cells metabolite, such as acetyl CoA, ATP, lactic acid, serine, SAM, and succinic acid, were reported to be closely related to a variety of inflammatory pathways.^92^, ^97^, ^98^, ^99^, ^100^ These metabolites could act as substrates for the related enzymes of epigenetic modification, such as histone acetylation, histone methylation, histone lactate, DNA methylation, and ATP‐mediated chromatin remodelling.^101^, ^102^ This crosstalk between metabolic reprogramming and epigenetic modification could affect the ‘open’ state of chromatin, to allow immune cells to accurately respond to the specific immune environment and make corresponding changes in function. In recent years, researchers have suggested that the integrated analysis of metabolism and epigenetics could be considered a new field: metabolo‐epigenomics.^96^

**FIGURE 4:**
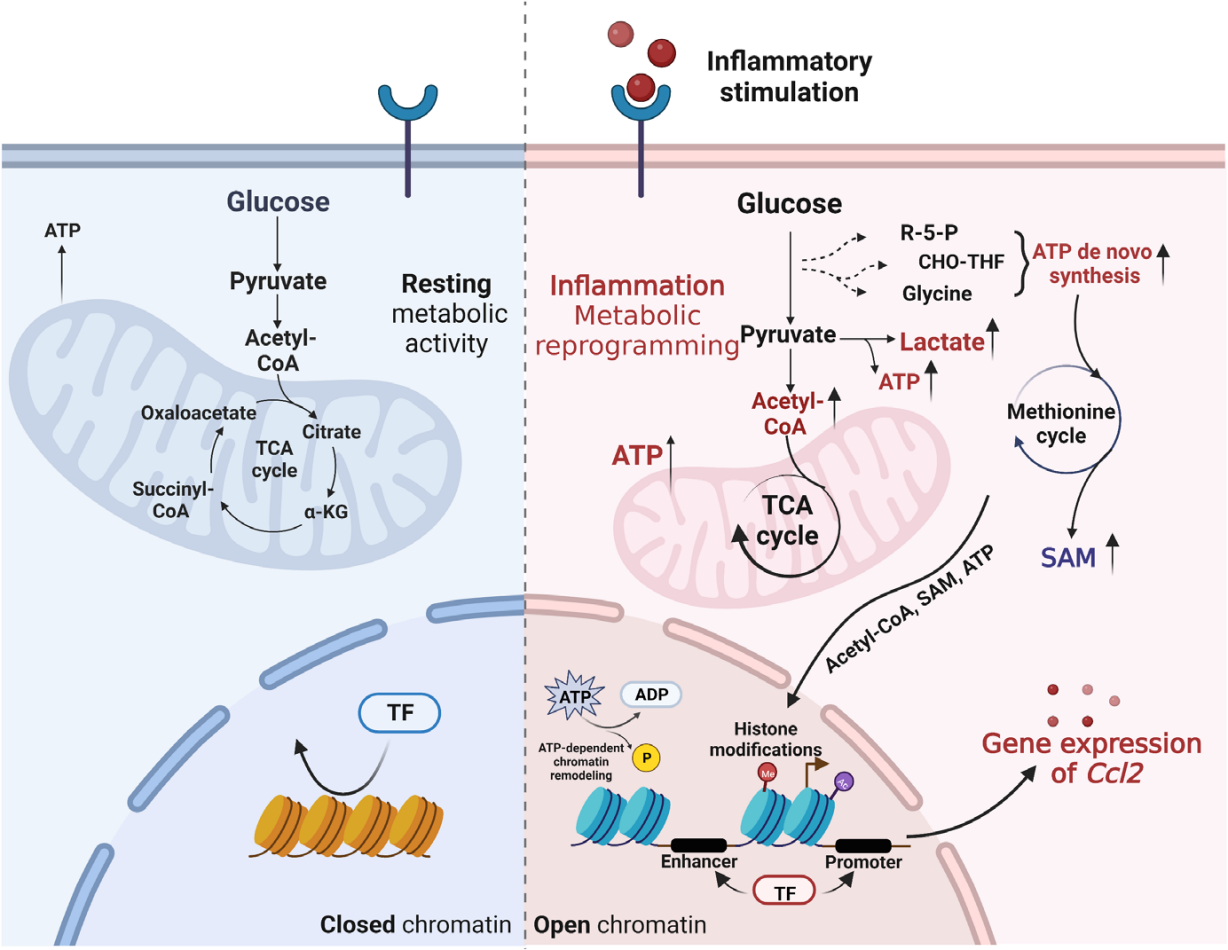
Metabolic‐epigenomics regulation of CCL2. Created with BioRender.com.

Interestingly, crosstalk between metabolism and epigenetics also plays an important role in CCL2 regulation in inflammatory environments. In the inflammatory state, immune cells undergo metabolic reprogramming, and multiple metabolic pathways, such as glycolysis and ‘de novo ATP generation’, significantly increase the generation of intracellular ATP.^98^, ^99^ Increased ATP will promote site‐specific ‘ATP‐dependent chromatin remodeling’ which consumes the energy of ATP hydrolysis to loosen the histone contact with DNA and thus promote transcription factor binding. In acute kidney injury, Brahma‐related gene 1 can continuously increase the binding of RNA polymerase II to the CCL2 gene by mediating chromatin remodelling, thus promoting hypoxic‐stress‐induced kidney proximal tubule CCL2 expression.^103^ Besides, in recent years, it has been found that LPS‐induced SAM production in the one‐carbon metabolism of macrophages significantly increased and then altered the methylation status of DNA and histone of target genes.^75^, ^104^ Exogenous supplements of methyl donors (such as folic acid, choline, etc.) could alleviate LPS‐induced macrophage activation and atherosclerosis by promoting SAM and DNA methylation.^75^, ^76^ Interestingly, it has been reported that under the induction of LPS, serine, as a methyl donor, could promote SAM synthesis through the methionine cycle. Consequently, SAM would promote the trimethylation of H3K36 and the expression of inflammatory factors of macrophages.^98^ The mechanism of the adverse effects of different methyl donors (folic acid and serine) on SAM formation through the methionine cycle remains to be further demonstrated. Overall, metabolism pathways play an important role in the functions of immune cells. A variety of metabolites regulate the epigenetic modification and transcriptional activity of CCL2. Therefore, metabolic pathways may provide new targets for inflammatory disease therapy (Figure 4).

## 6 CONCLUSION

Inflammation is a defensive response toward tissue injury, which plays an important role in maintaining tissue homeostasis.^105^ However, under the regulation of chemokines such as CCL2, a large number of immune cells gather at the inflammatory site, which leads to the activation of the chemoattractant‐cytokine network, resulting in the amplification and persistence of the inflammatory response.^14^ Due to its important role in the inflammatory cascade, CCL2 has become a therapeutic target for various inflammatory diseases such as insulin resistance, atherosclerosis, ischemic injury, and cancer. In recent years, more and more studies have reported the regulation of CCL2 mediated by epigenetic mechanisms, such as histone post‐transcriptional modifications (e.g., H3K27ac, H3K27me3), DNA methylation and ncRNA, and the interaction between epigenetic regulation and metabolic state of cells has been widely discussed. Therefore, reversible epigenetic changes may become a new idea for the treatment of inflammatory diseases. However, there are still many problems that need to be solved in epigenetic regulation. First, the cell type specificity of epigenetic modification, that is, the same epigenetic change acting on different cells may cause different effects on the target genes. Secondly, there may be a mutually reinforcing and self‐circulating complex epigenetic regulatory network between different epigenetic mechanisms. However, the role of this complex epigenetic network in inflammatory diseases remains to be further explored. A deeper exploration of epigenetic regulation is conducive to a more thorough understanding of inflammatory diseases, so as to provide a theoretical basis for the development of new targeted therapeutic drugs.

## AUTHOR CONTRIBUTIONS

Yi Liu conceived this review. Yingyi Chen and Siyan Liu contributed to search literatures and editing the manuscript. Lili Wu, Yitong Liu, Juan Du, and Zhenhua Luo contributed to the figure and the manuscript edition. The manuscript was edited and revised by Junji Xu and Lijia Guo. All authors read and approved the final manuscript. The author Yi Liu is the corresponding author. Yingyi Chen and Siyan Liu contributed equally to this work.

## CONFLICT OF INTEREST STATEMENT

The authors declare no conflicts of interest.

## Data Availability

Data sharing is not applicable to this article as no new data were created or analysed in this study.
